# 

**Published:** 1854-12

**Authors:** 


### CONTENTS OF THE DECEMBER NUMBER.

### ORIGINAL COMMUNICATIONS.

Page.

Art. I.—Statement of the Eruptive Disease as it prevailed in Hornellsville, Steuben Co., N. Y. By W. H. Renale, M. D., - - - - - - - - - - - - - - - - 385

Art. II.—Successful Amputation in a Case of Gangrene. By Drs. Jameson & Avery. - - 397

Art. III.—The Use of Sulphuric Acid. By N. L. North, M. D., - - - - - - - - 400

Art. IV—Minutes of the Proceedings of the Buffalo Med. Association. Reported by the Secretary, 405

Art. V.—Letter from Dr. Flint, - - - - - - - - - - - - - - - 412

Art. VI.—On the Nature, Signs, and Treatment of Childbed Fevers. By Chas. D. Meigs, M. D., - 419

Art. VII.—Auscultation and Percussion. Ay Dr. Joseph Skoda, - - - - - - 423

Art. VIII.—On the Diseases of the Interior Valley. By Daniel Drake, M. D., - - - - - 425

Art. IX.—Notes of M. Bernard's Lectures on the Blood. By Walter F. Atlee, M. D., - - - 427

Art. X.—Manual of Human Microscopical Anatomy. By A. Kolliker, - - - - - - 429

Art. XI.—Table of Meteorological Conditions in the City of Buffalo for October, 1854. By Sanford B. Hunt, M. D., - - - - - - - - - - - - - - - - 43[illegible]

Art. XII.—Monthly Record of Deaths in the City of Buffalo. By J. M. Newman, Health Physician, 432

### ECLECTIC DEPARTMENT.

Elkoplasty, or Anaplasty applied to the Treatment of Old Ulcers. By Frank H. Hamilton, M. D., 433

### EDITORIAL DEPARTMENT.

Dr. Cartwright on "Drapetomania." - - - - - - - - - - - - - - - 438

The Health of the City, - - - - - - - - - - - - - - - - - 442

Tracheotomy and Epilepsy, - - - - - - - - - - - - - - - - 443

On the Construction, etc., etc., of Hospitals for the Insane. By Thos. S. Kirkbride, M. D., - - 445

Transactions of the American Medical Association for 1854, - - - - - - - - 445

Transactions of the Med. Society of the State of Pennsylvania for 1854, - - - - - - 446

The Cholera in London, - - - - - - - - - - - - - - - 447

Mr. Spencer Wells on Gout, - - - - - - - - - - - - - - - 448

To Correspondents, - - - - - - - - - - - - - - - - - - 418

